# A Rational Method of Producing Local Anesthesia

**Published:** 1909-05-15

**Authors:** Hermann Prinz

**Affiliations:** St. Louis, Mo.


### A RATIONAL METHOD OF PRODUCING LOCAL ANESTHESIA.

BY HERMANN PRINZ, M.D., D.D.S., ST. LOUIS, MO.

According to more recent therapeutic conception it is generally recognized that a drug or a combination of drugs which simultaneously produces local anemia and inhibition of the sensory nerves in a circumscribed area of tissue is the logical solution of the question of local anesthesia. Certain important factors, however, relative to the physiological and physical action of the solution employed for hypodermic injections upon the cell, govern the successful application of such methods. It is of prime importance, therefore, to comply with the laws regulating the absorption of injected solution, viz: osmotic pressure.

THE PHYSIOLOGICAL ACTION OF ANESTHETICS.

If we separate two solutions of salt of different concentration by a permeable membrane, a continuous current of salt-and-water, results, which ceases only after equalization of the density of the two liquids, viz: when equal osmotic pressure—according to the Boyle-Van't Hoff law—is established. The current passes in both directions, drawing salt from the stronger to the weaker solution and water, vice versa, until osmotic equilibrium is obtained. The resultant solutions are termed isotonic (DeVries). Osmotic pressure is a physical phenomenon which is possessed by water and all aqueous solutions; it is dependent upon the number of molecules of salt present in the solution and upon their power of dissociation. In organized nature these osmotic interchanges play an important factor in regulating the tissue fluids of both animals and plants. The life of the cell depends upon the continuous passage of the fluids which furnish the nutrient materials, consisting of water, salt, and albumin. These chemicals are normally present in certain definite proportions. The membrane of the living cell is, however, only semipermeable, viz: the cell readily absorbs distilled water when surrounded therewith; it becomes macerated, loses its normal structure, and finally dies. If, on the other hand, the surrounding fluid be a highly concentrated salt solution, the solution absorbs water from the cell; no salt molecules enter into the cell body proper. The cell shrinks, and finally it also dies. This process of cell death is in general pathology referred to as necrobiosis.

A further important factor teaches us that all aqueous solutions which are isotonic possess the same freezing point, viz: all solutions possessing an equal freezing-point are equimolecular; they possess equal osmotic pressure. This law of physical chemistry has materially simplified the preparation of such solutions. The freezing point of human blood, lymph, serum, etc., has been found to equal, approximately, 0.55 C., which in turn corresponds to a 0.9 per cent. sodium chloride solution. Such a solution is termed a physiological salt solution. In the older works on physiology a 0.6 per cent. sodium chloride solution is referred to as a physiological salt solution; this solution corresponds to the density of the blood of a frog. A slight deviation above and below the normal percentage of the solid constituents is permissible. When physiological salt solution at body temperature is injected into the loose connective tissues under the skin in moderate quantities neither swelling nor shrinking of the cells as such occurs; a simple wheel is formed which soon dissappears, therefore no irritation results and in consequence no pain is felt.

Other similar bodies which are equally soluble in water act in the same manner, with the exception of the salts of the alkalie and earth metals, such as potassium or sodium bromid, for example. The latter substances produce intense physical irritation, followed, however, by prolonged anesthesia, and in consequence are termed by Liebreich "painful anesthetics." If, on the other hand, simple distilled water be injected, a superficial anesthesia only is produced; the injection itself is very painful and acts as a direct protoplasm poison by macerating the cell contents and resulting in the death of the cell. If distilled water approximately at the ratio of 10 drams to the pound of body weight be injected into dogs, they will succumb in a short time. The injection of higher concentrated salt solutions produces opposite effects; water is removed from the cells with more or less pronounced pain, also followed by superficial anesthesia. Several tissue disturbances result which may terminate in gangrene. Hypotonic solutions, then, cause swelling of the tissue, while hypertonic solutions produce shrinkage. These manifestations are proportionately the more intense the further the solution is removed from the freezing-point of the blood. Furthermore, hypotonic solutions as well as hypertonic solutions require much more time for their absorptions than isotonic solutions; the osmotic pressure has to be standardized to the surrounding fluid, viz, to the isotonic index of the tissue fluids. Local anemia or ischemia—viz, a temporary construction of circulation—prevents, as has been experimentally shown, the rapid absorption of fluids which are injected into the affected area.

The more important means to produce local anemia are —(1) The Esmarch elastic bandage. (2) The application of cold. (3) The extract of the suparenal capsule.

Some observers have maintained that local anemia as such produces anesthesia. This, however is not the case. It is merely an important means to confine the injected anesthetic to the anemic region and thus bring about a decreased absorption of the drug which increases and prolongs its actions. Conequently, the concentration of the anesthetic solution may be of a lower percentage, which of course lessens the danger of intoxication.

### SOME RECENT ANESTHETICS.

Within the last decade the active principle of the suprarenal capsule has demanded extensive comments in therapeutical literature. It has been isolated by a number of investigators under different names, viz, as epinephrin Abel (1897), suprarenin by Fuerth (1898), and adrenalin by Takimine and Aldrich (1901); and many other titles are given to this chemical, viz. paranephrin, suprarenalin, hemostasin, etc. Adrenalin is a grayish white powder, slightly alkaline in reaction and perfectly stable in dry form. It is sparingly soluble in cold and more soluble in hot water, and is insoluble in ether or alcohol; with acids it forms readily soluble salts. (I wish to be understood that whenever I refer to adrenalin in the following discussion, the hydrochlorid of the alkaloid of the suprarenal capsule is meant.) The preparation which is employed mostly for therapeutic purposes is a solution of adrenalin hydrochlorid in a physiological salt solution 1:1000, to which preservatives, such as small quantities of chloretone, thymol, etc., are added. Adrenalin solutions do not keep well—on exposure to air they are easily oxidized, becoming pink, then red, and finally brown; with this change of color the physiological properties of the adrenalin solution are destroyed. If the solution be further diluted it becomes practically worthless within a few days.

When adrenalin is injected into the tissues, even in extremely small doses, it temporarily raises the arterial blood pressure, acting as a powerful vaso-constrictor by stimulating the smooth muscular coat of the blood vesesls, and thus produces local anemia. Large doses finally reduce the blood pressure, and heart-failure results. The respiration at first quickly increases, but slows down and finally stops with the expiration. Its action is largely confined to the smooth muscle fibres of the peripheral vessels. Adrenalin is destroyed by the living tissue cells, the body ridding itself of the poison in some unknown manner. While adrenalin does not possess local anesthetic action, it increases very markedly the effect of certain anesthetics when they are injected into the tissues. It is claimed that secondary hemorrhage frequently follows after the anemia produced by the adrenalin has subsided, and that the tissues themselves suffer from the poisoning effects of the drug, resulting in gangrene. Such results are only produced by the injection of too large quantities of the drug, which by their deeper action close up the larger arteries. The prolonged anemia will give way to a dilatation of the blood vessels, and if the tissues are too long deprived of the circulation we are able to understand why sloughing may result. Small doses of adrenalin have no effect upon the tissues as such, or upon the healing of a wound. Palpitation of the heart, and muscle quivers, which occasionally were noticed in the early period of the use of the drug, were the direct result of too large doses. Recently adrenalin has been successfully prepared synthetically by Stolz. This new substance, of which a number of chemical modifications are known, possesses practically the same pharmacological action as the natural adrenalin. I will not enter into a discussion of the synthetical adrenalin at this moment, the experimental work being yet in its infancy. At some future period, however, I intend to refer to it more specifically.

For dental purposes, viz: for injection into the gum tissue, the dose should be limited to one drop of the adrenalin to each cubic centimeter of the anesthetic solution, five drops being approximately the maximum dose to be injected at one time.

### THE LOCAL ANESTHETICS.

Cocain, when injected into the tissues, produces typical local and general effects. Locally, it possesses a definite affinity for the peripheral nerves; it causes constriction of the smaller arteries, producing light anemia in the injected area with diminishing action of the leucocytes. However, different parts of the organism require different doses to bring about the same reaction. Upon mucous surfaces, paralysis of the sensory nerves is produced; the senses of touch and smell are temporarily inhibited. The blood as such and the circulation suffer little. If cocain in sufficient quantities is absorbed by the circulation, general manifestations are produced from bringing other tissues in close contact with the poison. The principal disturbances of the central nervous system make themselves known by vertigo, a very slight pulse, enlarged and staring pupils, and difficult respiration. Vomiting may occur; the throat feels dry. Intense excitement is followed by epileptiform spasms; finally, complete loss of sensation and mobility results, which terminates in death from cessation of respiration. The general character of the disturbances is closely related to that which occurs in chloroform or ether poisoning. The typical picture when the blood flowing through the central nervous system contains a sufficient quantity of the drug, even for a moment only, which is dangerous to this organ. No maximum dose can be positively established. This is equally true of cholcroform and ether when used for general anesthetic purposes. The many cases of so-called idiosyncrasy find an explanation in the too large doses which formerly were so frequently administered.

With our increased knowledge of the action of cocain upon the tissues and a proper technique of the injection, dangerous results are comparatively rare at present. No direct antidotes are known; the treatment of general intoxication is purely symptomatic. Anemia of the brain, which is of little consequence, may be readily overcome by placing the patient in a recumbent position or by complete inversion, if necessary. As a powerful dilator of the peripheral vessels the vapors of amyl nitrite are exceedingly useful; it is best administered by placing three to five drops of the fluid upon a napkin and holding it before the nostrils for inhalation. Flushing of the face and an increase in the frequency of the pulse follows almost momentarily. For convenience' sake, amyl nitrite may be procured in small glass pearls, holding the necessary quantity for one inhalation. Nausea may be remedied by administering small doses of peppermint, aromatic spirits of ammonia, or validol. The latter is a compound of menthol and valerianic acid and deserves special recommendation. To overcome the disturbances of respiration, quickly instituted artificial respiration is the alpha and omega of all methods of resuscitation; the only drug that has proved to be of value in this connection is strychnine in the form of sulfate or the nitrite in full doses by means of hypodermic injections.

The relative toxicity of a given quantity of cocain solution depends upon the concentration of the solution. Reclus and others have clearly demonstrated that a fixed quantity of cocain in a 5 per cent. or 10 per cent. solution is almost equally as poisonous as five times the same quantity in a ½ per cent. solution. From the extensive literature on the subject, we are safe in fixing the strength of the solution for dental purposes at 1 per cent. This quantity of cocain raises the freezing-point of distilled water just a little above 0.1 degree C. To obtain an isotonic solution corresponding to the freezing-point of the blood, 0.8 per cent. of sodium chlorid must be added. Having thus prepared a cocain solution which is equal to the blood in its osmotic pressure upon the cell walls, it is now necessary to aid the slightest vaso-constrictor power of the drug by the addition of a moderate quantity of adrenalin, thus increasing the confinement of the solution to the injected area by producing a deeper anemia for the twofold purpose—first, to act as a means of increasing the anesthetic effect of cocain, and second, to lessen its toxity upon the general system by slower absorption. As stated above, one drop of the adrenalin solution added to 1 c.c. of the isotonic cocain solution is sufficient to produce the desired effect.

### RECENT SUBSTITUTES FOR COCAIN.

Ever since the introduction of cocain into the materia medica for the purpose of producing local anesthesia, quite a number of substitutes have been placed before the profession, for which superiority in one respect or another is claimed over the original cocain. The more prominent members of this group are tropa-cocain, the eucains, accin, nirvanin, alypin, stovain, and novocain. None of these compounds with the exceptions of novocain has proved satisfactory for the purpose in view. The classical researches of Braun have established certain factors which are imperative relative to the value of a local anesthetic. The principal properties of such a chemical must correspond to the following claims: (1) In comparison to its local anesthetic value it must be less toxic than cocain. The difference of toxicity must be absolute, viz, the quantity of the drug necessary to produce the same anesthetic effect as a definite quantity of cocain must be less toxic to the same amount of body weight.

(2)—The chemical must be absolutely indifferent to the tissues when injected in more or less concentrated solutions. The progress of wound healing must not be interfered with by this solution. (3)—The chemical must be readily soluble in water, the solution must be comparatively stable, and it should be possible to sterilize it by simple means. (4)—The remedy must be tolerant to the addition of adrenalin without interfering with the vaso-constrictor power of the latter drug. (5)—When applied to mucous surfaces, ready penetration of the drug is necessary.

The limitation of this paper does not allow me to enter into a discussion of the pharmacological action of the drugs usually classified as local anesthetics. Let it suffice to state how the above-mentioned drugs fulfill the demands of Braun. Tropa-cocain is less poisonous but also less active than cocain, it completely destroys the action of adrenalin; the eucains partially destroy the adrenalin action, they are, comparatively speaking, equally as poisonous as cocain; accin is irritating to the tissues and much more poisonous than cocain; nirvanin possesses little anesthetic value; alypin and stovain are closely related, producing severe pain when injected, which occasionally results in gangrene.

NOVOCAIN.

Novocain alone fully corresponds to every one of the above claims. Its toxicity is about six to seven times less than cocain; it does not irritate in the slightest degree when injected, consequently no pain is felt from its injection *per se*; it is soluble in its own weight of water; it will combine with adrenalin in any proportion without interfering with the physiological action of the latter and it will be readily absorbed by the mucous membrane. The studies of Biberfeld and Braun brought to light another extremely interesting factor concerning the novocain-adrenalin combination. Both experimenters, working independently of each other, observed that the adrenalin anemia on the one hand and the novocain anesthesia on the other hand were markedly increased in their total effects upon the tissues. Consequently, a smaller quantity of this most happy combination is required to produce the same therapeutic effect as a larger dose of each individual drug alone would produce when injected separately. The injection of a solution of the combined drugs is precisely confined to the injected area; general effects are therefore rarely produced.

Novocain has been recently (1905), discovered by Prof. Einhorn. It is a synthetical product, representing the hydrochlorid of para-amino-benzoyl-diethylamin-ethanol; it appears in colorless needle shaped crystals, readily soluble in one part of water and thirty parts of alcohol. Its solution may be boiled without decomposition; it reacts neutral to litmus paper. In general, it is incompatible with caustic alkalies and carbonates and the alkaloid reagents. Novocain possesses the same anesthetic power as cocain upon peripheral nerves. In one-quarter per cent. solution it is sufficiently powerful to anesthetize even large nerves, viz.: It is equal in its anesthetic potency to any of the known local anesthetics. Pharmacologists (Biberfeld, Heineke and Laewen), have shown that novocain is about six to seven times less poisonous as compared with cocain.

As stated above, the relative toxicity of a given quantity of cocain in solution depends upon its concentration; this same peculiarity is not shared by novocain. The dose of novocain may be safely fixed at one-third of a grain for a single injection. For dental purposes a two per cent. solution is preferably employed; as much as three grains of a two per cent. solution in combination with adrenalin has been injected without any ill results. For the purpose of confining the injected novocain to a given area, the addition of adrenalin in small doses on account of its powerful vaso-constrictor action is admirably adapted. It is the important factor which prevents the ready absorption of both drugs and consequently largely nullifies poisonous results. An injection to ten drops of a two per cent. solution of novocain labially into the tissues produces a diffuse anesthesia lasting approximately twenty minutes; the same quantity with the addition of one drop of adrenalin chlorid solution increases the anesthetic period to over one hour and localizes the effect upon the injected area. In proof, the two solutions may be tinted with methylene blue and then injected into the subcutaneous tissue of the forearm; the colored areas are nicely outlined and can be tested by needle prickings.

A suitable solution of novocain for dental purposes may be prepared as follows:

| | |
|---|---|
| Novocain - - - - - | 10 grains. |
| Sodium chlorid - - - - - | 4 grains. |
| Distilled water - - - - | 1 fl. ounce. |
| Boil. | |

To each syringeful (30 minims), add two drops of adrenalin chlorid solution when used. Ready-made solutions of cocain, and equally so of novocain, will not keep when frequently exposed to the air; they irritate the tissues when injected, and frequently produce inflammatory infiltrations. A perfectly sterile solution may be made extemporaneously by dissolving the necessary amount of novocain-adrenalin in tablet form in a given quantity of boiled distilled water. A suitabel tablet may be prepared as follows:

| | |
|---|---|
| Novocain - - - - - | 1-3 grain. |
| Adrenalin hydrochlorid - - | 1-1500 grain. |
| Sodium chlorid - - - - - | $\frac{1}{8}$ grain. |

One tablet dissolved in 15 minims of sterile water makes a two per cent. solution of novocain ready for immediate use.

Solutions for hypodermic purposes should be made fresh when needed. A small glass dish with a pointed concave depression and a dropping-bottle constitute the simple outfit for such work. The dropping-bottle should hold one to two ounces. A very suitable one is made by the Whitall-Tatum Co., of Philadelphia, and may be bought in the drug shops. "A groove on one side of the neck of the bottle and a vent on the other connected with two grooves in the back of the stopper allow the contents to flow out drop by drop. A quarter turn of the stopper closes the bottle tightly.'' The water used for making the solution should be filtered, distilled water, and be boiled. The hypodermic solution can be made extemporaneously in twenty seconds: Place a tablet in the sterile glass dish, add 15 minims—which is approximately equal to 1 c.c.—of water, and to facilitate the solution, mash the tablet. It makes a half syringeful of a two per cent. solution of novocain.

### A SUITABLE HYPODERMIC SYRINGE.

A good hypodermic syringe which answers all dental purposes equally well is an important factor in the successful technique of the injection. The injection into the dense gum tissues frequently requires from 15 to 50 or even more pounds of pressure as registered by an interposed dynamometer, while in pressure anesthesia 100 to 200 or more pounds are frequently applied.

The selection of a suitable hypodermic syringe is largely a matter of choice; all-glass syringes, glass-barrel syringes, and all-metal syringes are the usual types found in the depots. If an all-glass syringe is preferred, the ''Sub-Q'' or the ''Luer'' syringe can be recommended. Glass-barrel syringes are not to be recommended for dental purposes, as they are too troublesome to keep in order. After testing most of the dental hypodermic syringes as offered in the dental depots within the last five years by means of the pressure gage and in clinical work, subjecting the syringe to a routine wear and tear, I find that the Manhattan all-metal non-corrosive platinoid syringe gives the best all-round service and I can conscientiously recommend it to my confreres. The syringe holds 30 minims (2 c.c). It is provided with a strong finger cross-bar and is extremely simple in construction. The piston consists of a plain metal rod without a thickened and ground piston end or packing. The piston rod is sufficiently long to allow about two inches of space between the finger rest and the piston top. This space is of importance; it allows the last drop of the fluid to be expelled under heavy pressure without tiring the hand. The packing consists of leather washers inserted at the screw joint. It is quickly removed and replaced if necessary. The washers for the piston and the needles are conveniently kept in the hollow piston top.

The hypodermic syringe requires careful attention. It is not necessary to sterilize it by boiling after each use unless contaminated with blood or pus. The simple repeated washing with alcohol and careful drying is sufficient. Readjust the cap, cover the piston rod with a thin film of vaselin, and place in position. If the syringe is boiled all the washers should be removed. Keep it in a covered glass or metal case; leather or felt lined boxes afford breeding-places for bacteria. Some operators prefer to constantly keep their syringe in an antiseptic solution when not in use; others prefer to place it in a special sterilizing bottle. A suitable sterilizing liquid for such purposes may be composed of—

| | |
|---|---|
| Cresol - - - - - - | 1 fl. dram |
| Alcohol - - - - - - | 1 fl. ounce |
| Water, enough to make - - - | 6 fl. ounces |

Dental hypodermic needles must be strong; they should be of reinforced steel, 27 B. & S. gage, and be provided with a short razor-edge point. Thicker needles cause unnecessary pain; finer needles are liable to break. The needle itself should measure a quarter of an inch. For infiltrating larger surfaces, one-half to one-inch needles are necessary. Curved needles are essential in reaching the posterior teeth. For pressure anesthesia needles are required. They may be bought at the depots or quickly prepared by grinding off the steel needle at its point of reinforcement. The sterile needles should be kept in a well-corked glass tube, and should always be sterilized by boiling after each use in a two per cent. lysol or cresol solution in a teaspoon or test tube, dried with the hot air syringe and immediately transferred to a sterile glass bottle. Novccain is precipitated from its solution by sodium carbonate; if soda is used for sterilizing purposes the syringe or needles must be washed with sterile water. The sterile needles should not be touched with the fingers again. The customary wire insertion is unnecessary.—*Dental Era.*

